# Microbiota, the immune system, black moods and the brain—melancholia updated

**DOI:** 10.3389/fnhum.2014.00720

**Published:** 2014-09-15

**Authors:** Lesley E. Smythies, John R. Smythies

**Affiliations:** ^1^Department of Medicine (Gastroenterology), University of Alabama at BirminghamBirmingham, AL, USA; ^2^Department of Psychiatry, University of Alabama at BirminghamBirmingham, AL, USA; ^3^Department of Psychology, Center for Brain and Cognition, University of California, San DiegoSan Diego, CA, USA

**Keywords:** microbiota, MDD, inflammation, stress, leaky gut hypothesis, exosomes, psychobiotics, gut-brain networks

## Introduction

There is now abundant evidence that the immune system and the brain have close functional interactions in both directions. This review focuses on the influence of the microbiota (the bacteria resident in the gut) on brain function in major depressive disorder (MDD) and related syndromes.

The gut and the central nervous system communicate partly through the autonomic nervous system (ANS), consisting of the sympathetic (i.e., the splanchnic nerves) and the parasympathetic (i.e., the vagus nerve and the sacral parasympathetic pelvic nerves) nervous system. The brain integrates inputs from the digestive tract by a central autonomic network that includes the hypothalamus, limbic system and cerebral cortex with output to the ANS and hypothalamic-pituitary-adrenal gland (HPA) stress axis (Bonaz, 2013). There are also extensive chemical signaling systems via the blood.

## Gut-brain interactions in MDD

Altered immune responses and increased inflammation have been demonstrated in MDD (Herbert and Cohen, 1993; Licinio and Wong, 1999; Miller et al., 2009a,b; Dowlati et al., 2010; Maes, 2011; Raedler, 2011; Frodl and Amico, 2014). These stressful reactions are mediated in part by pro-inflammatory cytokines (and other signaling molecules) that originate in the intestinal mucosa and interact with the hypothalamic–pituitary–adrenal gland (HPA) stress axis and neurotransmitter metabolism at various levels. Risk factors include psychosocial stressors, diets with high levels of refined carbohydrates and saturated fatty acids, low physical activity, smoking, obesity and vitamin D deficiency (Berk et al., 2013).

Stress-induced pro-inflammatory cytokines can elicit profound changes in brain function and behavior, which include the initiation of depressive symptoms, such as sad mood, anhedonia, fatigue, psychomotor retardation, and social-behavioral withdrawal (Slavich and Irwin, 2014). High levels of interleukin (IL)-6, C-reactive protein, tumor necrosis factor (TNF)-α, and neopterin are found in patients suffering from MDD (Müller, 2014). Indeed, cytokine receptors are present in neurons and glial cell populations in discrete brain regions (Arisi, 2014), including the hypothalamus, nucleus accumbens, hippocampus, thalamus, cortex, and cerebellum. Moreover, cytokines have direct and indirect effects on neurotransmitter storage and release by microglia cells and astrocytes. This includes the tryptophan kynurenine metabolic pathway. This key enzyme that degrades serotonin—indoleamine 2, 3-dioxygenase—is driven by pro- and anti-inflammatory cytokines. Neuronal levels of serotonin play a key role in MDD. Moreover, neuroactive kynurenines, such as kynurenic acid and quinolinic acid, act on the glutamatergic neurotransmission as N-methyl-D-aspartate antagonists and agonists, respectively. Severe infections and autoimmune disorders are lifetime risk factors for MDD (Müller, 2014). Stress may even contribute to a lasting pro-inflammatory state. Further supportive evidence is provided by the therapeutic benefit in MDD of anti-inflammatory medications, such as cyclo-oxygenase-2 inhibitors, TNF-α antagonists and others, and the anti-inflammatory and immunomodulatory intrinsic effects of antidepressants (Müller, 2014).

Brain function encoding emotion also depends on interoceptive signals such as visceral pain. In irritable bowel syndrome (IBS), which is characterized by chronic recurrent abdominal pain, disorder of brain-gut function may be associated with corticotropin-releasing hormone (CRH) and/or serotonin, since administration of CRH exacerbates the visceral sensorimotor response, whereas inhibition of CRH or serotonin with their respective antagonists or antidepressants is protective (Fukudo, 2013). Moreover, studies of congential insensitivity to pain with anhidrosis (CIPA) show that the Nerve Growth Factor–TrkA system is essential for the establishment of a neural network for interoception and homeostasis that may underlie “gut feelings” (Indo, 2009). The complex interplay of neuro-endocrine-immune top-down and bottom-up pathways in IBS, closely related to neuropsychiatric disorders, is well reviewed by Stasi et al. (2012) and in functional dyspepsia by Van Oudenhove and Aziz (2013).

## The “Leaky Gut” hypothesis

In recent papers Berk et al. (2013), Lucas and Maes (2013), and McCusker and Kelley (2013) have presented the “leaky gut” hypothesis, which suggests that a key mechanism involved in MDD may be increased permeability of the gut epithelium, leading to the abnormal intestinal absorption of luminal bacteria that, under normal circumstances, would be restricted to the gut lumen by the epithelial barrier. Bacteria carry on their surfaces a variety of pathogen-associated molecular patterns (PAMPs) that are recognized by specific receptors (PRRs) located both on the surface membrane (e.g., toll-like receptors (TLRs)) and in the cytoplasm (e.g., nucleotide-binding oligomerization domain (Nod)-like receptors) of cells belonging to the innate immune system, primarily macrophages and dendritic cells (DCs) (Leulier and Lemaitre, 2008; McCusker and Kelley, 2013). If the epithelial barrier is circumvented, bacteria [or molecules from them, such as lipopolysaccharides (LPS)] penetrating the intestinal mucosa may bind to their cognate TLRs (in particular TLR-4), driving NF-κ β activation, stimulating the expression of pro-inflammatory cytokines, including IL-1 and TNF-α, and facilitating the synthesis of arachidonic-acid derivates (Feng et al., 1995; Verstrepen et al., 2008), all of which are implicated in MDD.

Evidence supporting this hypothesis includes the following findings. First, chronic mild stress increases the level of bacterial LPS in the circulation of rats, driving activation of the TLR-4 pathway on blood monocytes (Gárate et al., 2011, 2013), and production of immunoglobulins against LPS is increased in MDD (Maes et al., 2008; Maes and Kubera, 2012; Maes et al., 2013). Second, exposure to stress activates TLR-4 signaling in the frontal cortex of mice (Gárate et al., 2011), resulting in oxidative and nitrosative neuronal damage. Lastly, Kéri et al. (2014) reported that expression of TLR-4 RNA and protein, NF-κ B and 16S RNA (a subunit of intestinal microbiota) were raised in the plasma of patients with MDD compared to healthy controls, and their magnitude correlated with the severity of the clinical symptoms.

TLRs are also expressed on neurons and are thought to regulate neurodevelopment, dendritic cell growth and behavior in mice (Liu et al., 2013). Additionally, some bacteria can also directly activate nociceptive neurons by evoking calcium flux and action potentials without involving TLRs (Chiu et al., 2013), a mechanism that involves bacterial N-formylated peptides and the pore-forming toxin alpha-hemolysin. These bacterial toxins and cytokines (or signals from them) are transported to the brain by nervous pathways (e.g., vagal signaling via the n. tractus solitarius to the hypothalamus and amygdala) and humeral transport via the lymph and blood. Sako et al. (2005) studied c-FOS expression in the brain in response to peripheral administration of bacterial CpG, reporting increased numbers of c-FOS-positive cells in the paraventricular nucleus of the hypothalamus, the nucleus of the tractus solitarius (NTS) and the area postrema, independently of the afferent vagus nerve input. A possible carrier for bacterial toxins, including CpG-DNA, from the gut lumen into the brain may be exosomes (see further below).

The pertinent questions now relate to the nature of the mechanism (i) by which microbiotic toxins and their effects are transmitted from the gut to the brain and (ii) what specific mechanisms in the brain are affected. Kéri et al. (2014) stipulate that this involves bacterial translocation or “the presence of various damage-associated molecular patterns.”

## Exosomes

We can now ask what is the mechanism by which bacterial toxins cross the intestinal endothelium. One established mechanism is provided by sub-epithelial intestinal DCs that insert dendrites between epithelial cells to harvest luminal bacteria or soluble products from the gut lumen (McDole et al., 2012). We suggested previously that this cargo may be transported within exosomes (small lipoprotein vesicles that contain protein, nucleic acids, sugars and lipids), and transferred from the DCs to T cells in the draining lymph nodes, where they can modulate inflammatory responses (Smythies and Smythies, 2014). We now add a further detail to this hypothesis, whereby PSA-labeled exosomes released by bacteria in the gut lumen may also be taken up by intestinal DCs and carried to the lymph nodes, to activate T-cell immune responses (Shen et al., 2012). Thus, T cells can receive epigenetic material from gut bacteria, either by direct endocytosis, or via afferent exosomes. Moreover, exosomes entering the general circulation, via the lymph and blood, can be transmitted to the brain with subsequent effects on the brain's inflammatory system as we described earlier. Lastly, exosomes themselves influence the properties of the blood-brain barrier to facilitate their passage into the brain. This involves changes in the membrane electric resistance and capacitance (Ridder et al., 2014).

But how would psychological stress activate this pathway? Since exosome traffic between cells is usually bidirectional, one mechanism could be that stress, via the CRH—HPA pathway, could alter the epigenetic content of exosomes exported from DCs into the lumen and taken up by gut bacteria, thereby altering bacterial profiles, and modulating the epigenetic loads of exosomes (particularly miRNAs) subsequently exported from these bacteria and taken up by intestinal DCs. Indeed, olfactory bulbectomy mice demonstrate chronic depression- and anxiety-like behaviors associated with elevated central CRH expression and increased c-Fos activity, serotonin levels, and motility in the colon (Park et al., 2013). These changes are accompanied by an altered intestinal microbial profile. Central CRH administration produces similar changes in behavior and motility and altered the microbiota profile in the colon. Remarkably, the alteration of the microbial profile in the colon produced by acute social stress is required for the latter to increase circulating cytokine levels (Bailey et al., 2011). The monumental complexities of the epigenetic regulation (particularly by DNA methylation) of stress related functions in the brain have been well reviewed by Klengel et al. (2014). For a general review of the role of epigenetic mechanisms in information processing in the brain see Edelstein et al. (2014).

Bretz et al. (2013) report that exosomes, isolated from various body fluids that were internalized by THP-1 cells, induced the production of IL-1β, TNF-α, and IL-6. The signaling pathways involved a fast triggering of NF-κ B and a delayed activation of the transcription factor STAT3. The initial production of IL-6 was necessary for the STAT3 activation. Exosomal signaling was TLR-dependent as the knockdown of TLR2 and TLR4 blocked NF-κ B and STAT3 activation.

## Psychobiotics

Therapeutic modifications of the gut microbiota may be achieved by feeding certain bacteria such as some Lactobacilli. Alterations in the central expression of GABA are implicated in the pathogenesis of anxiety and depression (Bravo et al., 2011). These workers report that chronic administration of *Lactobacillus rhamnosus* induces region specific changes in the expression of GABA (B1b) mRNA in the brain with increases in the cingulate and prelimbic cortices and reductions in the hippocampus, amygdala and locus coeruleus. It also reduced GABA (Aa2) mRNA expression in the prefrontal cortex and amygdala but increased this measure in the hippocampus. *Lactobacillus rhamnosus* feeding also reduced stress-induced corticosterone levels and anxiety and depression-related behaviors. These findings were abolished following vagotomy. Thus, the vagus must carry this signal from the gut to the brain. The use of psychobiotics (such as *Bifidobacterium infantis*) in relieving anxiety and depression associated with IBS is reviewed by Dinan and Cryan (2013). Ait-Belgnaoui et al. (2014) report that pretreatment with a combination of *Lactobacillus helveticus* and *Bifidobacterium longum* attenuated stress-induced activation of the HPA axis and the autonomic nervous system, reducing cFos expression in several brain areas, as well as reducing stress-induced hippocampal neurogenesis and expression changes in genes in the hypothalamus concerned in synaptic plasticity. The therapeutic applications of differential bacterial colonizations is reviewed by Buffie and Pamer (2013).

The powerful effect on the microbiota during *Clostridium difficile* (CD) infections may affect the mood in addition to the distress caused by the symptoms. The use of antidepressant medications increases the risk of developing CD infection (Rogers et al., 2013). Oral administration of vancomycin significantly impacts host physiology by decreasing intestinal microbiota diversity, bile acid dehydroxylation and peripheral insulin sensitivity (Vrieze et al., 2014).

## Conclusion

This paper reviews the present status of the “leaky gut” hypothesis relating to MDD and adds some details of the molecular and cellular mechanisms that may be involved. This hypothesis suggests that a key mechanism involved in MDD may be increased gut permeability, abnormal intestinal absorption of gram-negative bacteria, and subsequent activation of the TLR/NF-κ B pathway in innate immune cells of the gut mucosa, or on neurons themselves. This activation induces the release of pro-inflammatory cytokines with a deleterious effect on a number of brain mechanisms involved in stress. We suggest that exosomes may play a prominent part in this process.

### Conflict of interest statement

The authors declare that the research was conducted in the absence of any commercial or financial relationships that could be construed as a potential conflict of interest.
